# Crystal structure of (3*R*)-3-benzyl-4-[(*tert*-but­oxy­carbon­yl)amino]­butanoic acid

**DOI:** 10.1107/S1600536814019497

**Published:** 2014-09-03

**Authors:** Karol Jędrzejczak, Małgorzata Szczesio, Monika Oracz, Stefan Jankowski, Marek L. Główka

**Affiliations:** a Institute of Organic Chemistry, Faculty of Chemistry, Lodz University of Technology, Żeromskiego 116, Łódź, Poland; b Institute of General and Ecological Chemistry, Faculty of Chemistry, Lodz University of Technology, Żeromskiego 116, Łódź, Poland

**Keywords:** crystal structure, butanoic acid, monosubstituted γ-amino acids, hydrogen bonding

## Abstract

The characteristic feature of the title mol­ecule, C_16_H_23_NO_4_, is the *syn* configuration of the partially double amide C—N bond [C—N—C—O torsion angle = −14.8 (2)°]. The crystal packing is determined by inter­molecular O—H⋯O and N—H⋯O hydrogen bonds, which link the mol­ecules into a double-chain structure extending along [010].

## Related literature   

The title enanti­omeric compound was synthesized according to Loukas *et al.* (2003 ▶) and Felluga *et al.* (2008 ▶). For related structures, see: Pihko & Koskinen (1998 ▶); Jimeno *et al.* (2011 ▶). For solution conformation of oligomers based on monosubstituted γ-amino acids, see: Guo *et al.* (2012 ▶); Kang & Byun (2012 ▶). For amino acid analysis by HPLC after derivatization with Marfey’s reagent, see: Marfey (1984 ▶).
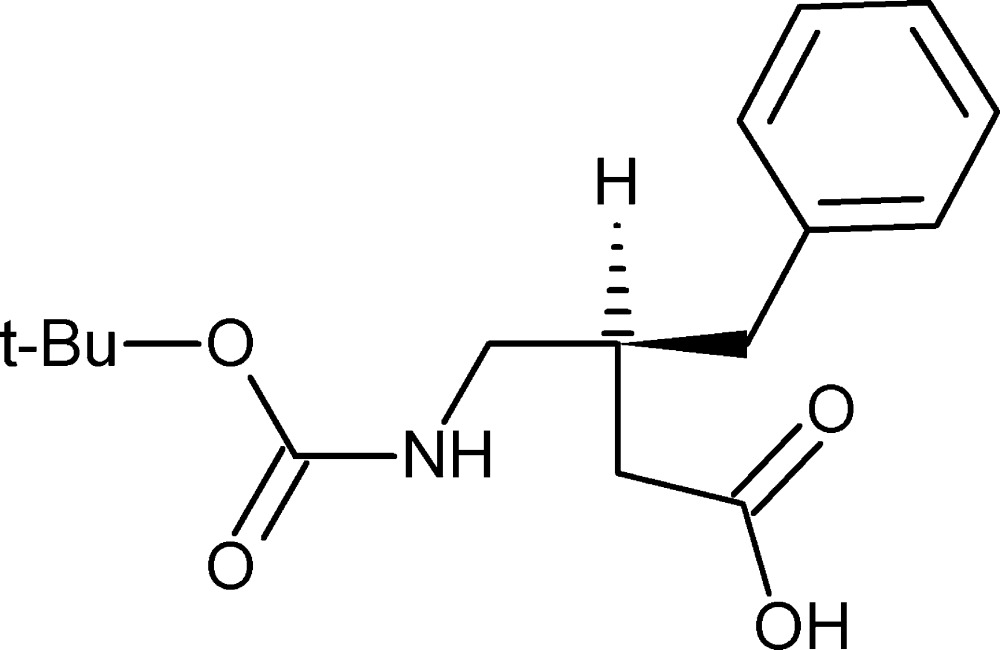


## Experimental   

### Crystal data  

C_16_H_23_NO_4_
*M* *_r_* = 293.35
Monoclinic, 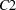

*a* = 19.5872 (12) Å
*b* = 6.5263 (4) Å
*c* = 14.7598 (9) Å
β = 120.846 (2)°
*V* = 1619.89 (17) Å^3^
*Z* = 4
Cu *K*α radiation
μ = 0.70 mm^−1^
*T* = 100 K
0.4 × 0.04 × 0.04 mm

### Data collection  

Bruker SMART APEX CCD diffractometer
Absorption correction: multi-scan (*SADABS*; Sheldrick, 2003 ▶) *T*_min_ = 0.738, *T*_max_ = 0.973
8769 measured reflections
2880 independent reflections
2805 reflections with *I* > 2σ(*I*)
*R*_int_ = 0.036

### Refinement  

*R*[*F* ^2^ > 2σ(*F* ^2^)] = 0.029
*wR*(*F* ^2^) = 0.073
*S* = 1.06
2880 reflections
197 parameters
1 restraint
H atoms treated by a mixture of independent and constrained refinement
Δρ_max_ = 0.16 e Å^−3^
Δρ_min_ = −0.18 e Å^−3^
Absolute structure: Flack (1983 ▶), 1138 Friedel pairs
Absolute structure parameter: 0.05 (15)

### 

Data collection: *APEX2* (Bruker, 2005 ▶); cell refinement: *SAINT-Plus* (Bruker, 2008 ▶); data reduction: *SAINT-Plus*; program(s) used to solve structure: *SHELXS97* (Sheldrick, 2008 ▶); program(s) used to refine structure: *SHELXL97* (Sheldrick, 2008 ▶); molecular graphics: *PLATON* (Spek, 2009 ▶) and *Mercury* (Macrae *et al.*, 2006 ▶); software used to prepare material for publication: *PLATON*.

## Supplementary Material

Crystal structure: contains datablock(s) I, New_Global_Publ_Block. DOI: 10.1107/S1600536814019497/gk2614sup1.cif

Structure factors: contains datablock(s) I. DOI: 10.1107/S1600536814019497/gk2614Isup2.hkl

Supporting information file. DOI: 10.1107/S1600536814019497/gk2614Isup3.cml

**Fig. 1.**
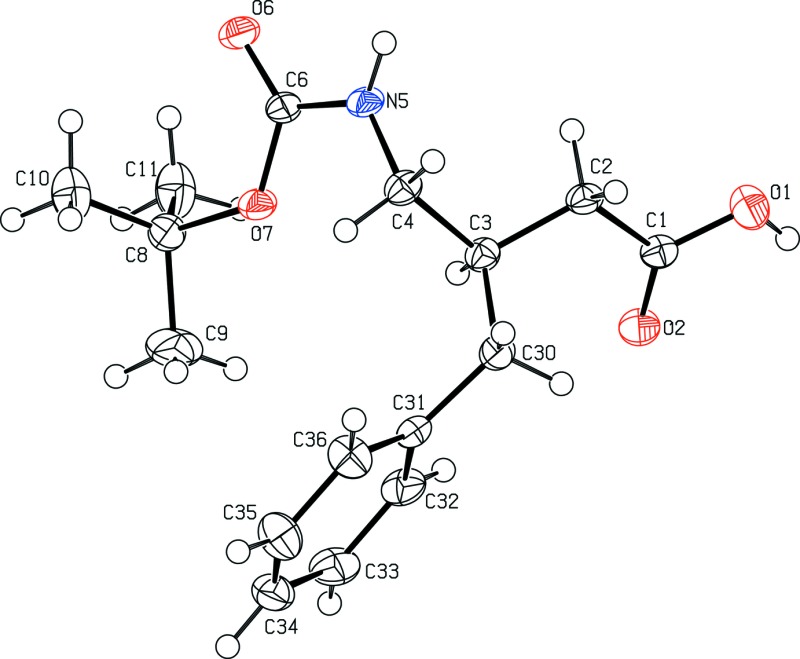
The molecular structure with displacement ellipsoids drawn at the 50% probability level.

. DOI: 10.1107/S1600536814019497/gk2614fig1.tif
The mol­ecular structure with displacement ellipsoids drawn at the 50% probability level.

**Fig. 2.**
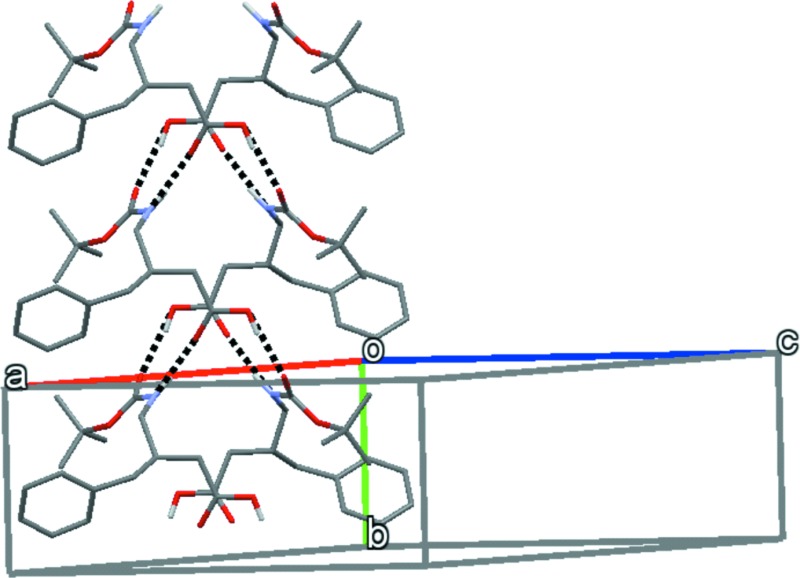
Packing of the title compound viewed along the [101] direction.

. DOI: 10.1107/S1600536814019497/gk2614fig2.tif
Packing of the title compound viewed along the [101] direction.

CCDC reference: 938020

Additional supporting information:  crystallographic information; 3D view; checkCIF report

## Figures and Tables

**Table 1:** Hydrogen-bond geometry (Å, °)

*D*—H⋯*A*	*D*—H	H⋯*A*	*D*⋯*A*	*D*—H⋯*A*
O1—H1⋯O6^i^	0.82	1.83	2.6368 (15)	170
N5—H5⋯O2^ii^	0.846 (18)	2.131 (18)	2.8856 (16)	148.2 (15)

Symmetry codes: (i) 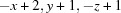; (ii) 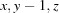.

## supplementary crystallographic information

### S1. Comment

γ-Amino acids are important components of α,γ-peptide hybrids, which are resistant towards enzymatic degradation and, as a result, display useful biological activity, including antibiotic, antiviral and anticancer properties. The acids are also important elements of foldamers. In comparison with the α-amino acids, they show significant flexibility due to the two additional single bonds between the carboxylic and amine functions. Still, their oligomers form well defined conformations in solutions, in particular helical ones in the case of monosubstituted γ-amino acids (Guo *et al.*, 2012, Kang *et al.*, 2012). Thus, the structures and common conformations of γ-amino acids and their derivatives are of interest. The molecular structure is shown in Figure 1. The crystal packing is determined by intermolecular N5—H···O2 and O1—H···O6 hydrogen bonds, which organize the molecules into infinite double chains parallel to the [010] direction (Fig.2). The geometrical parameters of the hydrogen bonds are listed in Table 1.

### S2. Experimental

(3*R*)-4-((*tert*-Butoxycarbonyl)amino-)-3-benzyl-butanoic acid was obtained from racemic (±)-3-aminomethyl-4-phenylbutanoic acid hydrochloride, which was synthesized following earlier published procedure (Felluga *et al.*, 2008), with some modifications. Ethyl (±)-3-nitromethyl-4-phenylbutanoate was hydrolyzed and then hydrogenated using 10% Pd/C to get acid, which was transformed into Boc-derivative and purified by crystallization from ethyl acetate/hexane.

Enantiomeric resolution of racemic (±)-3-aminomethyl-4-phenylbutanoic acid (1 g) was achieved by crystallization from ethyl acetate (110 mL) in the presence of (*S*)-(-)-methylbenzylamine (0.41 g). The solution was left for 24 h at +5°C for crystallization, which was repeated four times to obtain (3*S*)-4-((*tert*-butoxycarbonyl)amino-)-3-benzyl-butanoic acid (0.151 g) with ee = 97.4 %. (*R*)-(+)-Methylbenzylamine (0.17 g) was applied to the mother liquor after the first crystallization of (3*S*)-4-((*tert*-butoxycarbonyl)amino-)-3-benzyl-butanoic acid ammonium salt. Three subsequent crystallizations led to (3*R*)-(-)-4-((*tert*-butoxycarbonyl)amino-)-3-phenyl-pentanoic acid (0.196 g) with ee = 98.1 %. Acids were recovered from ethyl acetate solution using 1M NaHSO_4_ solution.

The enantiomeric purity was determined according to the known procedure using N_α_-(2,4-dinitro-5-fluorophenyl)-L-valinamide as derivating reagent (Marfey, 1984). Sample of enantiomer (5 mg) was dissolved in TFA – dichloromethane (1:1), the solution was shaken for 10 min, then solvents were removed by evaporation. The residue was dissolved in CH_2_Cl_2_ and the solvent was removed again. This procedure was repeated five times to remove TFA completely. The dry residue was dissolved in 0.2 M NaHCO_3_ to obtain 0.05 M solutions (0.5 mL) of (3R)-4-amino–3-benzyl-butanoic acid. Mixture of 0.05 M aqueous solution of deprotected amino acid (25 µL), 0.2 N NaHCO_3_ (50 µL), 1% solution of N_α_-(2,4-dinitro-5-fluorophenyl)-L-valine amide in acetone (50 µL) and 75 µL of acetone was shaken for 1 minute and then placed in a water bath for 45 min at 45°C. Then mixture was shaken again for 30 sec, 0.1M HCl (170 µL) and acetone (75 µL) were added. A yellowish solution was analysed by HPLC (Vydac column C8 (4.6 x 25 cm), gradient 40 - 80, detection at 340 nm), diastereomeric derivative of (3*R*)-4-amino-3-benzyl-butanoic acid was detected at 12.67 min retention time.

Single crystals were obtained by recrystallization from acetonitrile at room temperatute.

### S3. Refinement

All H atoms were located in difference Fourier maps but finally their positions were determined geometrically, except H5 that was freely refined. H atoms were refined as riding on their carriers with C—H= 0.95 Å for aromatic CH groups, 0.97 Å for CH_2_ groups, 0.96 Å for methyl groups and N—H = 0.86 Å for the amide group, and with *U*_iso_(H) = 1.2U_eq_(C,N), except for methyl group where *U*_iso_(H) = 1.5U_eq_(C). The absolute structure was known from the synthetic procedure and is confirmed by the Flack parameter of 0.05 (15).

### Crystal data

**Table:**

C_16_H_23_NO_4_	*F*(000) = 632
*M**_r_* = 293.35	*D*_x_ = 1.203 Mg m^−^^3^
Monoclinic, *C*2	Cu *K*α radiation, λ = 1.54178 Å
Hall symbol: C 2y	Cell parameters from 3858 reflections
*a* = 19.5872 (12) Å	θ = 3.5–64.2°
*b* = 6.5263 (4) Å	µ = 0.70 mm^−^^1^
*c* = 14.7598 (9) Å	*T* = 100 K
β = 120.846 (2)°	Needle, colourless
*V* = 1619.89 (17) Å^3^	0.4 × 0.04 × 0.04 mm
*Z* = 4	

### Data collection

**Table:**

Bruker SMART APEX CCD diffractometer	2880 independent reflections
Radiation source: fine-focus sealed tube	2805 reflections with *I* > 2σ(*I*)
Graphite monochromator	*R*_int_ = 0.036
ω scan	θ_max_ = 72.4°, θ_min_ = 3.5°
Absorption correction: multi-scan (*SADABS*; Sheldrick, 2003)	*h* = −24→24
*T*_min_ = 0.738, *T*_max_ = 0.973	*k* = −7→8
8769 measured reflections	*l* = −18→18

### Refinement

**Table:**

Refinement on *F*^2^	Secondary atom site location: difference Fourier map
Least-squares matrix: full	Hydrogen site location: inferred from neighbouring sites
*R*[*F*^2^ > 2σ(*F*^2^)] = 0.029	H atoms treated by a mixture of independent and constrained refinement
*wR*(*F*^2^) = 0.073	*w* = 1/[σ^2^(*F*_o_^2^) + (0.0236*P*)^2^ + 0.6631*P*] where *P* = (*F*_o_^2^ + 2*F*_c_^2^)/3
*S* = 1.06	(Δ/σ)_max_ = 0.001
2880 reflections	Δρ_max_ = 0.16 e Å^−^^3^
197 parameters	Δρ_min_ = −0.18 e Å^−^^3^
1 restraint	Absolute structure: Flack (1983), 1138 Friedel pairs
Primary atom site location: structure-invariant direct methods	Absolute structure parameter: 0.05 (15)

### Special details

**Table:**

Geometry. All e.s.d.'s (except the e.s.d. in the dihedral angle between two l.s. planes) are estimated using the full covariance matrix. The cell e.s.d.'s are taken into account individually in the estimation of e.s.d.'s in distances, angles and torsion angles; correlations between e.s.d.'s in cell parameters are only used when they are defined by crystal symmetry. An approximate (isotropic) treatment of cell e.s.d.'s is used for estimating e.s.d.'s involving l.s. planes.
Refinement. Refinement of *F*^2^ against ALL reflections. The weighted *R*-factor *wR* and goodness of fit *S* are based on *F*^2^, conventional *R*-factors *R* are based on *F*, with *F* set to zero for negative *F*^2^. The threshold expression of *F*^2^ > σ(*F*^2^) is used only for calculating *R*-factors(gt) *etc*. and is not relevant to the choice of reflections for refinement. *R*-factors based on *F*^2^ are statistically about twice as large as those based on *F*, and *R*- factors based on ALL data will be even larger.

### Fractional atomic coordinates and isotropic or equivalent isotropic displacement parameters (Å^2^)

**Table:**

	*x*	*y*	*z*	*U*_iso_*/*U*_eq_	
C1	0.89784 (7)	0.5954 (2)	0.40885 (10)	0.0213 (3)	
C2	0.88937 (8)	0.3833 (2)	0.44237 (10)	0.0220 (3)	
H2A	0.9309	0.2966	0.4459	0.026*	
H2B	0.8386	0.3268	0.3891	0.026*	
C3	0.89425 (7)	0.3775 (2)	0.54933 (10)	0.0211 (3)	
H3	0.9405	0.4574	0.6005	0.025*	
C4	0.90258 (7)	0.1581 (2)	0.58971 (10)	0.0226 (3)	
H4A	0.8936	0.1582	0.6485	0.027*	
H4B	0.8612	0.0753	0.5341	0.027*	
C6	1.04195 (8)	0.0711 (2)	0.72350 (10)	0.0215 (3)	
C8	1.08942 (8)	0.2534 (2)	0.89211 (11)	0.0285 (3)	
C9	1.05139 (13)	0.4342 (4)	0.91403 (14)	0.0596 (6)	
H9A	1.0502	0.5499	0.8731	0.089*	
H9B	1.0818	0.4680	0.9877	0.089*	
H9C	0.9981	0.3992	0.8952	0.089*	
C10	1.09383 (10)	0.0664 (3)	0.95540 (12)	0.0392 (4)	
H10A	1.0413	0.0302	0.9395	0.059*	
H10B	1.1265	0.0960	1.0293	0.059*	
H10C	1.1165	−0.0457	0.9375	0.059*	
C11	1.17061 (10)	0.3096 (3)	0.90952 (12)	0.0390 (4)	
H11A	1.1938	0.1918	0.8967	0.058*	
H11B	1.2045	0.3549	0.9809	0.058*	
H11C	1.1649	0.4176	0.8619	0.058*	
C30	0.81864 (8)	0.4716 (2)	0.53904 (10)	0.0234 (3)	
H30A	0.8038	0.5900	0.4930	0.028*	
H30B	0.7760	0.3723	0.5048	0.028*	
C31	0.82452 (7)	0.5377 (2)	0.64118 (10)	0.0212 (3)	
C32	0.86972 (9)	0.7077 (2)	0.69501 (12)	0.0290 (3)	
H32	0.9003	0.7720	0.6717	0.035*	
C33	0.86995 (9)	0.7831 (3)	0.78276 (13)	0.0346 (3)	
H33	0.9000	0.8985	0.8171	0.041*	
C34	0.82581 (9)	0.6883 (3)	0.81985 (11)	0.0318 (3)	
H34	0.8254	0.7405	0.8782	0.038*	
C35	0.78253 (9)	0.5154 (3)	0.76924 (12)	0.0348 (4)	
H35	0.7537	0.4484	0.7944	0.042*	
C36	0.78199 (8)	0.4414 (3)	0.68080 (11)	0.0297 (3)	
H36	0.7525	0.3247	0.6473	0.036*	
N5	0.97942 (6)	0.06104 (18)	0.62403 (9)	0.0218 (2)	
H5	0.9795 (9)	−0.034 (3)	0.5849 (12)	0.026*	
O1	0.88632 (7)	0.59996 (17)	0.31251 (8)	0.0335 (3)	
H1	0.8915	0.7177	0.2976	0.050*	
O2	0.91355 (6)	0.74770 (16)	0.46349 (8)	0.0286 (2)	
O6	1.10102 (6)	−0.03990 (16)	0.75856 (7)	0.0281 (2)	
O7	1.03178 (5)	0.21689 (15)	0.77878 (7)	0.0261 (2)	

### Atomic displacement parameters (Å^2^)

**Table:**

	*U*^11^	*U*^22^	*U*^33^	*U*^12^	*U*^13^	*U*^23^
C1	0.0191 (6)	0.0193 (7)	0.0236 (6)	−0.0012 (5)	0.0095 (5)	−0.0024 (5)
C2	0.0246 (6)	0.0160 (6)	0.0247 (6)	0.0011 (5)	0.0121 (5)	−0.0020 (5)
C3	0.0216 (6)	0.0175 (7)	0.0217 (6)	−0.0008 (5)	0.0093 (5)	−0.0006 (5)
C4	0.0227 (6)	0.0170 (7)	0.0256 (6)	−0.0016 (5)	0.0106 (5)	−0.0009 (5)
C6	0.0293 (7)	0.0125 (6)	0.0242 (6)	−0.0002 (5)	0.0147 (5)	−0.0012 (5)
C8	0.0341 (7)	0.0233 (8)	0.0208 (6)	0.0009 (6)	0.0088 (6)	−0.0044 (6)
C9	0.0666 (12)	0.0553 (13)	0.0349 (9)	0.0212 (10)	0.0103 (8)	−0.0206 (9)
C10	0.0449 (9)	0.0405 (10)	0.0286 (7)	−0.0090 (8)	0.0163 (7)	0.0027 (7)
C11	0.0421 (9)	0.0372 (10)	0.0275 (7)	−0.0131 (7)	0.0105 (7)	0.0005 (7)
C30	0.0237 (6)	0.0215 (7)	0.0233 (6)	0.0016 (5)	0.0107 (5)	0.0007 (5)
C31	0.0203 (6)	0.0167 (7)	0.0240 (6)	0.0037 (5)	0.0095 (5)	0.0021 (5)
C32	0.0345 (7)	0.0156 (7)	0.0400 (8)	−0.0024 (6)	0.0214 (7)	0.0001 (6)
C33	0.0384 (8)	0.0207 (8)	0.0414 (8)	−0.0041 (6)	0.0181 (7)	−0.0102 (6)
C34	0.0327 (7)	0.0338 (9)	0.0268 (7)	0.0042 (6)	0.0137 (6)	−0.0057 (6)
C35	0.0343 (7)	0.0409 (10)	0.0330 (8)	−0.0079 (7)	0.0201 (6)	−0.0033 (7)
C36	0.0296 (7)	0.0290 (8)	0.0308 (7)	−0.0097 (6)	0.0156 (6)	−0.0069 (6)
N5	0.0270 (6)	0.0122 (6)	0.0244 (5)	0.0004 (4)	0.0117 (5)	−0.0030 (4)
O1	0.0551 (7)	0.0190 (6)	0.0296 (5)	−0.0092 (5)	0.0241 (5)	−0.0031 (4)
O2	0.0384 (5)	0.0164 (5)	0.0290 (5)	−0.0035 (4)	0.0159 (4)	−0.0049 (4)
O6	0.0299 (5)	0.0220 (5)	0.0292 (5)	0.0074 (4)	0.0129 (4)	−0.0008 (4)
O7	0.0302 (5)	0.0195 (5)	0.0227 (5)	0.0052 (4)	0.0094 (4)	−0.0039 (4)

### Geometric parameters (Å, º)

**Table:**

C1—O2	1.2159 (17)	C10—H10A	0.9600
C1—O1	1.3209 (16)	C10—H10B	0.9600
C1—C2	1.507 (2)	C10—H10C	0.9600
C2—C3	1.5322 (17)	C11—H11A	0.9600
C2—H2A	0.9700	C11—H11B	0.9600
C2—H2B	0.9700	C11—H11C	0.9600
C3—C4	1.5272 (19)	C30—C31	1.5144 (18)
C3—C30	1.5373 (18)	C30—H30A	0.9700
C3—H3	0.9800	C30—H30B	0.9700
C4—N5	1.4634 (17)	C31—C32	1.388 (2)
C4—H4A	0.9700	C31—C36	1.3894 (19)
C4—H4B	0.9700	C32—C33	1.383 (2)
C6—O6	1.2318 (16)	C32—H32	0.9300
C6—O7	1.3332 (16)	C33—C34	1.384 (2)
C6—N5	1.3476 (17)	C33—H33	0.9300
C8—O7	1.4809 (16)	C34—C35	1.379 (2)
C8—C10	1.512 (2)	C34—H34	0.9300
C8—C9	1.516 (2)	C35—C36	1.387 (2)
C8—C11	1.520 (2)	C35—H35	0.9300
C9—H9A	0.9600	C36—H36	0.9300
C9—H9B	0.9600	N5—H5	0.846 (18)
C9—H9C	0.9600	O1—H1	0.8200
			
O2—C1—O1	122.74 (13)	C8—C10—H10C	109.5
O2—C1—C2	124.47 (11)	H10A—C10—H10C	109.5
O1—C1—C2	112.79 (11)	H10B—C10—H10C	109.5
C1—C2—C3	113.68 (11)	C8—C11—H11A	109.5
C1—C2—H2A	108.8	C8—C11—H11B	109.5
C3—C2—H2A	108.8	H11A—C11—H11B	109.5
C1—C2—H2B	108.8	C8—C11—H11C	109.5
C3—C2—H2B	108.8	H11A—C11—H11C	109.5
H2A—C2—H2B	107.7	H11B—C11—H11C	109.5
C4—C3—C2	111.29 (11)	C31—C30—C3	115.96 (10)
C4—C3—C30	108.53 (11)	C31—C30—H30A	108.3
C2—C3—C30	109.89 (10)	C3—C30—H30A	108.3
C4—C3—H3	109.0	C31—C30—H30B	108.3
C2—C3—H3	109.0	C3—C30—H30B	108.3
C30—C3—H3	109.0	H30A—C30—H30B	107.4
N5—C4—C3	115.21 (11)	C32—C31—C36	117.71 (13)
N5—C4—H4A	108.5	C32—C31—C30	119.88 (12)
C3—C4—H4A	108.5	C36—C31—C30	122.28 (12)
N5—C4—H4B	108.5	C33—C32—C31	121.01 (14)
C3—C4—H4B	108.5	C33—C32—H32	119.5
H4A—C4—H4B	107.5	C31—C32—H32	119.5
O6—C6—O7	124.44 (12)	C32—C33—C34	120.58 (14)
O6—C6—N5	124.22 (12)	C32—C33—H33	119.7
O7—C6—N5	111.34 (11)	C34—C33—H33	119.7
O7—C8—C10	109.69 (12)	C35—C34—C33	119.14 (14)
O7—C8—C9	101.12 (11)	C35—C34—H34	120.4
C10—C8—C9	112.02 (15)	C33—C34—H34	120.4
O7—C8—C11	110.82 (12)	C34—C35—C36	120.05 (14)
C10—C8—C11	111.52 (13)	C34—C35—H35	120.0
C9—C8—C11	111.23 (16)	C36—C35—H35	120.0
C8—C9—H9A	109.5	C35—C36—C31	121.45 (14)
C8—C9—H9B	109.5	C35—C36—H36	119.3
H9A—C9—H9B	109.5	C31—C36—H36	119.3
C8—C9—H9C	109.5	C6—N5—C4	123.71 (11)
H9A—C9—H9C	109.5	C6—N5—H5	117.4 (11)
H9B—C9—H9C	109.5	C4—N5—H5	116.2 (11)
C8—C10—H10A	109.5	C1—O1—H1	109.5
C8—C10—H10B	109.5	C6—O7—C8	122.65 (10)
H10A—C10—H10B	109.5		
			
O2—C1—C2—C3	5.74 (18)	C32—C33—C34—C35	1.1 (2)
O1—C1—C2—C3	−174.26 (11)	C33—C34—C35—C36	−1.6 (2)
C1—C2—C3—C4	−168.26 (10)	C34—C35—C36—C31	0.1 (2)
C1—C2—C3—C30	71.50 (14)	C32—C31—C36—C35	1.9 (2)
C2—C3—C4—N5	70.78 (14)	C30—C31—C36—C35	−174.09 (13)
C30—C3—C4—N5	−168.17 (11)	O6—C6—N5—C4	165.44 (13)
C4—C3—C30—C31	76.50 (15)	O7—C6—N5—C4	−14.81 (18)
C2—C3—C30—C31	−161.60 (11)	C3—C4—N5—C6	89.89 (15)
C3—C30—C31—C32	70.93 (17)	O6—C6—O7—C8	−3.7 (2)
C3—C30—C31—C36	−113.17 (15)	N5—C6—O7—C8	176.54 (11)
C36—C31—C32—C33	−2.4 (2)	C10—C8—O7—C6	−63.08 (17)
C30—C31—C32—C33	173.69 (14)	C9—C8—O7—C6	178.48 (15)
C31—C32—C33—C34	1.0 (2)	C11—C8—O7—C6	60.48 (18)

### Hydrogen-bond geometry (Å, º)

**Table:**

*D*—H···*A*	*D*—H	H···*A*	*D*···*A*	*D*—H···*A*
O1—H1···O6^i^	0.82	1.83	2.6368 (15)	170
N5—H5···O2^ii^	0.846 (18)	2.131 (18)	2.8856 (16)	148.2 (15)

Symmetry codes: (i) −*x*+2, *y*+1, −*z*+1; (ii) *x*, *y*−1, *z*.

## References

[bb1] Bruker (2005). *APEX2* Bruker AXS Inc., Madison, Wisconsin, USA.

[bb2] Bruker (2008). *SAINT-Plus* Bruker AXS Inc., Madison, Wisconsin, USA.

[bb3] Felluga, F., Pitacco, G., Valentin, E. & Venneri, C. D. (2008). *Tetrahedron Asymmetry*, **19**, 945–955.

[bb4] Flack, H. D. (1983). *Acta Cryst.* A**39**, 876–881.

[bb5] Guo, L., Zhang, W., Guzei, I. A., Spencer, L. C. & Gellman, S. H. (2012). *Tetrahedron*, **68**, 4413–4417.

[bb6] Jimeno, C., Pericas, M. A., Wessel, H. P., Alker, A. & Muller, K. (2011). *ChemMedChem*, **6**, 1792–1795. 10.1002/cmdc.201100220 21805644

[bb7] Kang, Y. K. & Byun, B. J. (2012). *Biopolymers*, **97**, 1018–1025. 10.1002/bip.22119 22987592

[bb8] Loukas, V., Noula, C. & Kokotos, G. (2003). *J. Pept. Sci.* **9**, 312–319. 10.1002/psc.458 12803497

[bb9] Macrae, C. F., Edgington, P. R., McCabe, P., Pidcock, E., Shields, G. P., Taylor, R., Towler, M. & van de Streek, J. (2006). *J. Appl. Cryst.* **39**, 453–457.

[bb10] Marfey, P. (1984). *Carlsberg Res. Commun.* **49**, 591–596.

[bb11] Pihko, P. M. & Koskinen, A. M. P. (1998). *J. Org. Chem.* **63**, 92–98. 10.1021/jo971167m 11674047

[bb12] Sheldrick, G. M. (2003). *SADABS* University of Göttingen, Germany.

[bb13] Sheldrick, G. M. (2008). *Acta Cryst.* A**64**, 112–122. 10.1107/S0108767307043930 18156677

[bb14] Spek, A. L. (2009). *Acta Cryst.* D**65**, 148–155. 10.1107/S090744490804362X PMC2631630 19171970

