# Supplementary figures and images for: Crystal structure of (3R)-3-benzyl-4-[(tert-but­oxy­carbon­yl)amino]­butanoic acid
Source: Acta Crystallogr Sect E Struct Rep Online. 2014 Sep 3;70(Pt 10):o1081–2. doi: 10.1107/S1600536814019497 (PMC4257155; doi:10.1107/S1600536814019497)

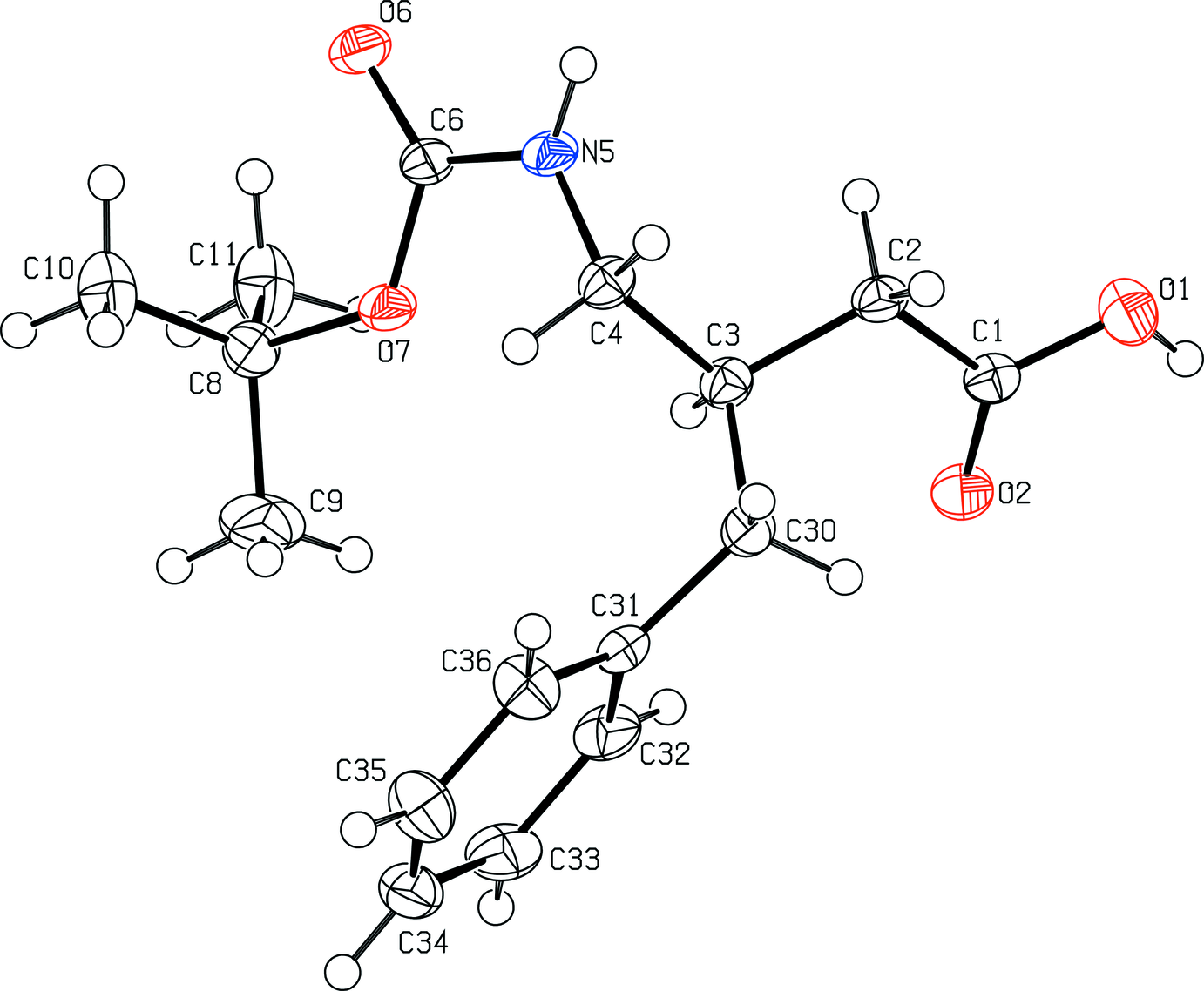

Supplement: Supplementary file 4 [file e-70-o1081-fig1.tif]

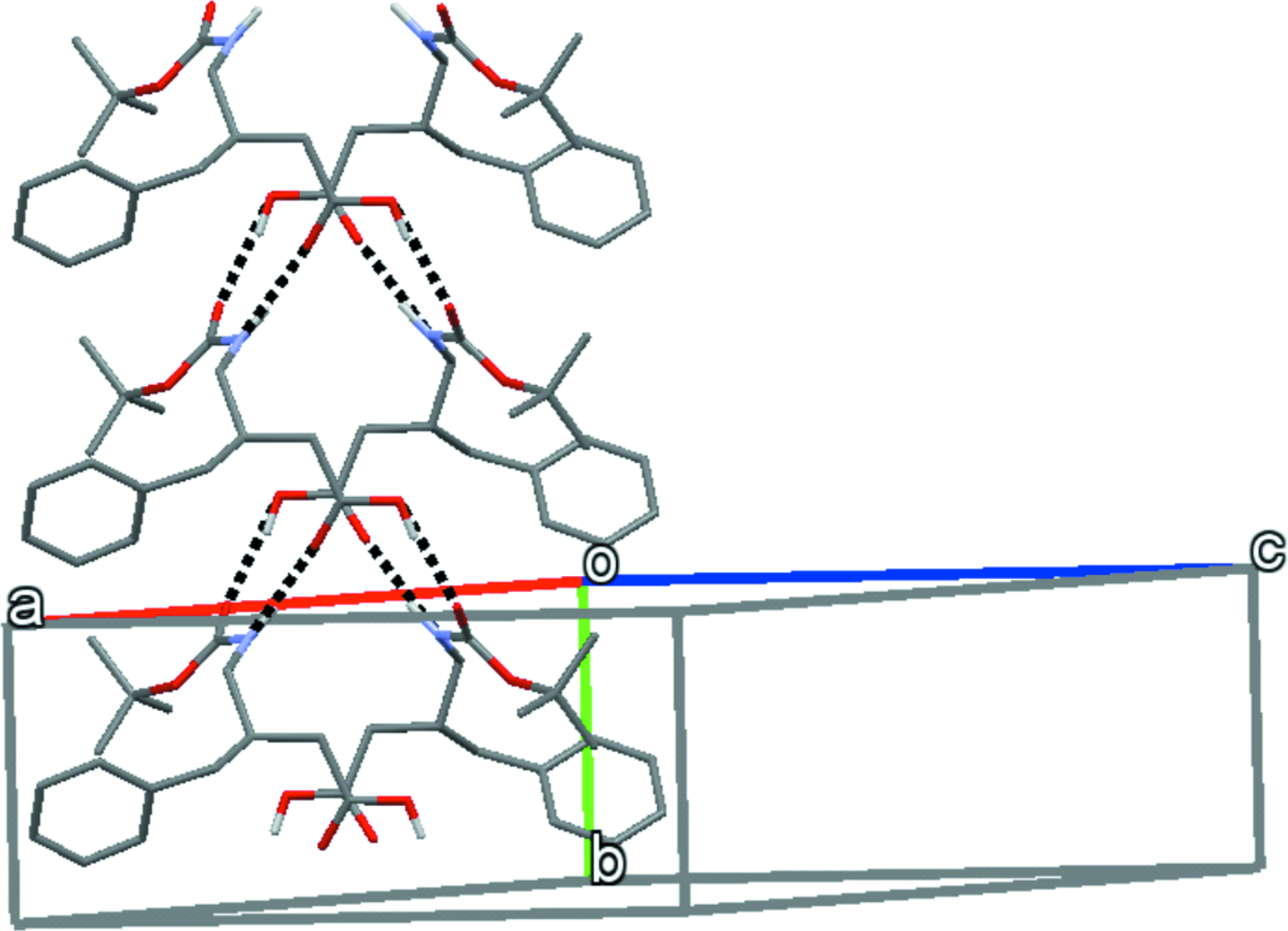

Supplement: Supplementary file 5 [file e-70-o1081-fig2.tif]
